# Benign Metastatic Leiomyoma Presenting as a Hemothorax

**DOI:** 10.1155/2013/504589

**Published:** 2013-07-17

**Authors:** Anna M. Ponea, Creticus P. Marak, Harmeen Goraya, Achuta K. Guddati

**Affiliations:** ^1^Division of Pulmonary and Critical Care Medicine, Montefiore Hospital, Albert Einstein College of Medicine, Yeshiva University, New York, NY 10467, USA; ^2^Department of Internal Medicine, Jacobi Medical Center, Albert Einstein College of Medicine, Yeshiva University, New York, NY 10461, USA; ^3^Department of Internal Medicine, Massachusetts General Hospital, Harvard Medical School, Harvard University, 50 Fruit Street, Boston, MA 02114, USA

## Abstract

Uterine leiomyomas have been reported to metastasize to various organs including the lungs, skeletal muscles, bone marrow, peritoneum, and heart. They may present with symptoms related to the metastases several years after hysterectomy. These tumors regress after menopause, and it is rare to detect active tumors in postmenopausal women. Despite their ability to metastasize, they are considered to be benign due to the lack of anaplasia. Pulmonary benign metastasizing leiomyoma is usually detected in the form of pulmonary nodules incidentally on imaging. Tissue biopsy of these nodules is required to identify them as benign metastasizing leiomyomas. Immunohistochemical analysis and molecular profiling may further help detect any malignant transformation in it. Untreated pulmonary benign metastasizing leiomyoma may result in the formation of cystic structures, destruction of lung parenchyma, and hemothorax and may cause respiratory failure. Surgical resection and hormonal therapy help prevent progression of this disease and provide an avenue for a cure.

## 1. Introduction

Uterine leiomyomas are benign tumors which affect up to 80% of females in their lifetime [1]. Family history, nulliparity, obesity, and race have been established as risk factors for development of uterine leiomyoma [2, 3]. They are more prevalent in women of African descent and cause infertility in up to 3% of patients [4, 5]. Metastasis of uterine leiomyoma was first described by Steiner in 1939 [6]. Uterine leiomyomas are considered to be benign but have been reported to metastasize to various organ including the lymph nodes, bones, skeletal muscles, bone marrow, nerves, peritoneum, retroperitoneum, heart, mediastinum, and lungs [7–13]. Histologically, they consist of dense nodules with cells and extracellular material arranged in a trabecular pattern punctuated by foci of cystic degeneration and microcalcifications. Microscopically, the trabecular pattern consists of spindle-shaped smooth muscle cells with abundant eosinophilic cytoplasm and elongated nuclei. However, several subtypes exist: benign metastasizing leiomyoma (BML) which is most often detected in lungs as nodules and consists of densely packed smooth muscle cells; epithelioid leiomyoma, consisting of polygonal cells; cellular leiomyoma, consisting of smooth muscles and collagen; vascular leiomyoma, consisting of abundant blood vessels; leiomyoma with tubules which consists of tubular structures; lipoleiomyoma, consisting of smooth muscle cells and adipocytes; myxoid leiomyoma, consisting of smooth muscles interspersed in myxoid substance; atypical leiomyoma, consisting of atypical cells distributed in extracellular matrix [14].

Metastatic foci of leiomyoma have been discovered up to 24 years after hysterectomy for benign leiomyoma of the uterus and the average age of presentation is 48 years [15, 16]. BML has been postulated to be a result of hematogenous spread from a benign uterine leiomyoma. It is currently unclear if hematogenous spread is an endogenous process characteristic of the primary disease or if it is facilitated by procedures such as dilatation and curettage. BML is considered benign despite its ability to metastasize because of the lack of mitotic figures and anaplasia. Pulmonary BML is often seen in premenopausal women but is rarely seen in postmenopausal women. This case report describes the clinical presentation, pathological features, and clinical course of a patient who was diagnosed with pulmonary BML after she was found to have a hemothorax.

## 2. Case Summary

The patient is a 64-year-old lady with a past medical history significant for hypertension, hyperlipidemia, hypothyroidism, and diabetes who presented with rapidly progressing dyspnea on exertion for the past two weeks. This was accompanied by a new onset progressive orthopnea and right-sided back pain of the same duration. She denied cough, hemoptysis, fever/chills, chest pain, and leg swelling. She had seen her primary care physician recently and was prescribed a trial of albuterol and was scheduled for an echocardiogram. Notably, her primary care physician documented clear lungs bilaterally, and the patient reported no improvement in her dyspnea with albuterol. She denied recent travel and had no history of a positive purified protein derivative (PPD) test. Two years ago, she was noted to have increased abdominal girth, and imaging showed uterine and ovarian masses. She subsequently underwent total abdominal hysterectomy with bilateral salpingo-oophorectomy, and pathology showed benign leiomyomas. During the same time, she was also diagnosed with multiple lung nodules and underwent a CT-guided biopsy which showed atypical cells and smooth muscle and was inconclusive. In the same year, she underwent colonoscopy which showed a benign polyp and a mammogram which showed dense irregular tissue in her right breast. Due to the discomfort experienced due to the CT-guided biopsy, she refused to have repeat evaluation of her lung nodules and was lost to follow-up. Her family history was significant for colon cancer in her mother. She worked in the office of a pediatrician and was an active smoker but denied consumption of alcohol or usage of intravenous drugs. Her medications included lisinopril, hydrochlorothiazide, levothyroxine, and an albuterol inhaler.

**Figure 1:**
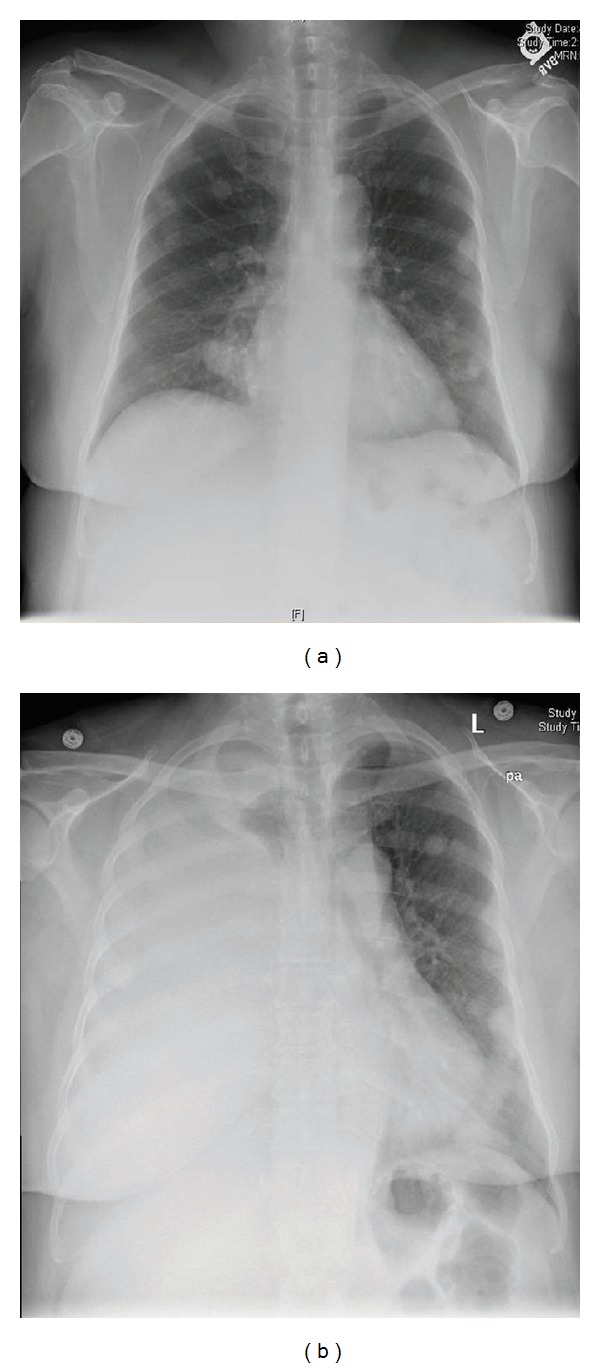
(a) CXR taken two years before presentation. (b) CXR at the time of presentation shows a white-out of the right hemithorax.

**Figure 2:**
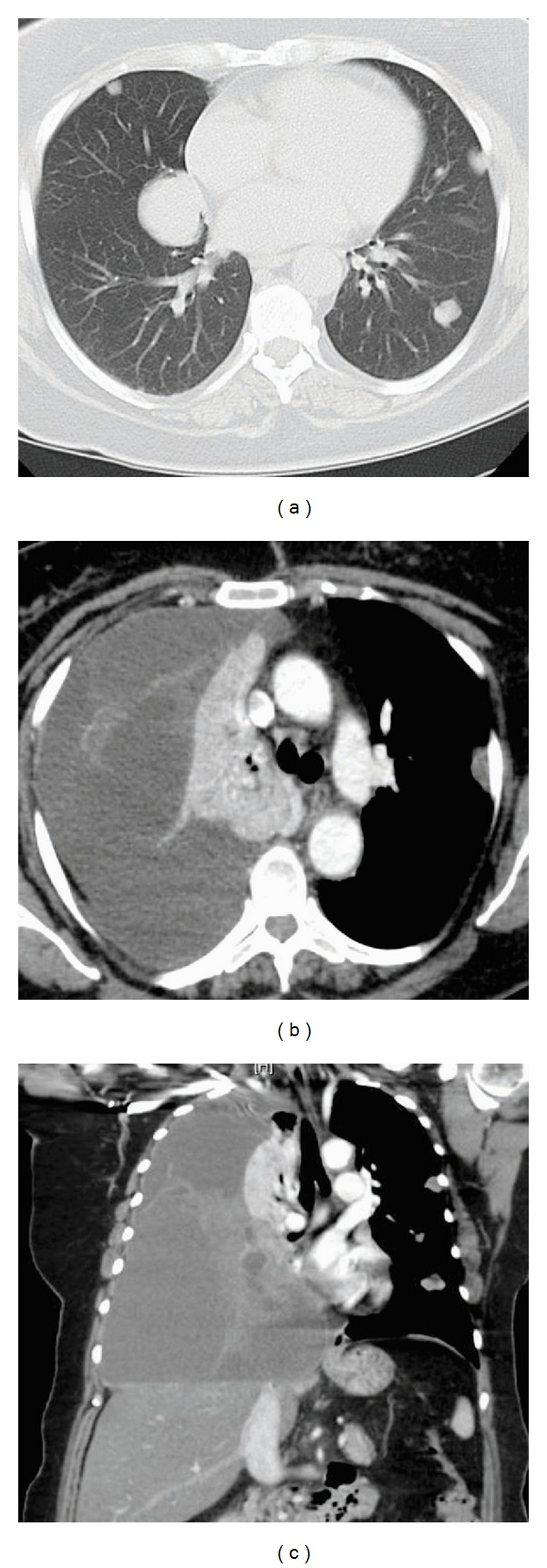
(a) Transverse section of thorax by CT before presentation. (b) Transverse section of thorax by CT at presentation showing fluid-filled right hemithorax with an irregular mass. (c) Coronal section of thorax by CT at presentation showing fluid-filled right hemithorax with an irregular mass.

**Figure 3:**
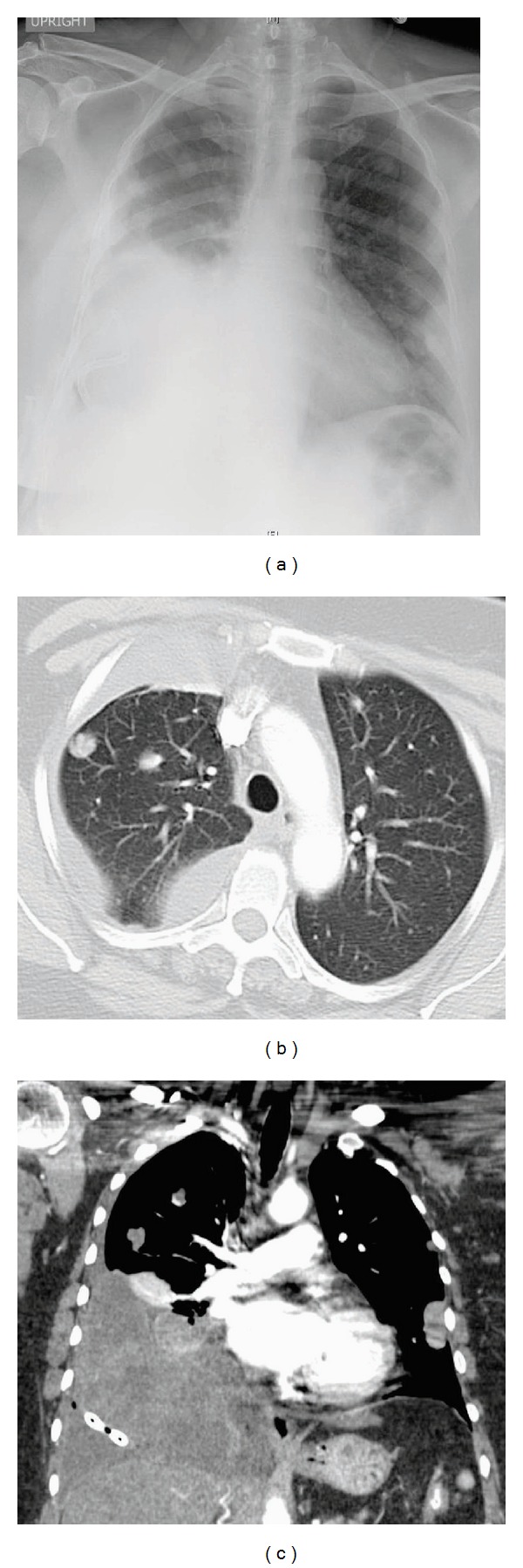
(a) CXR showing residual lower lobe collection of fluid after insertion of chest tube. (b) Transverse section of thorax by CT showing restoration of lung parenchyma in the upper lobe of the right lung. (c) Coronal section of thorax by CT showing a significant collection of fluid in the middle and lower right lobes of the lung.

**Figure 4:**
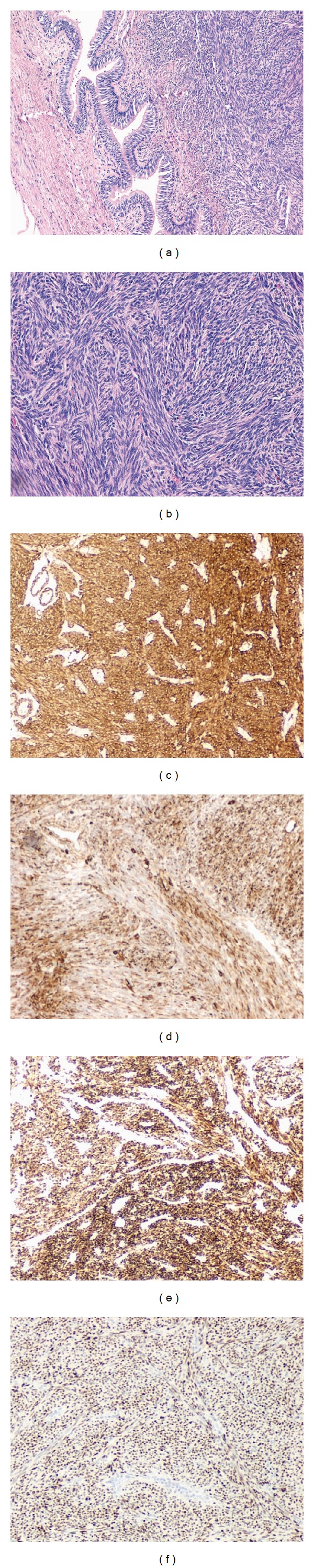
(a) Hematoxylin and Eosin (H&E) stain at low magnification showing two foci of BML. (b) H&E stain at a higher magnification showing trabeculated arrangement of spindle cells. (c), (d), (e), and (f) Immunohistochemical staining positive for SMA, vimentin, desmin, and CK 7.

**Figure 5:**
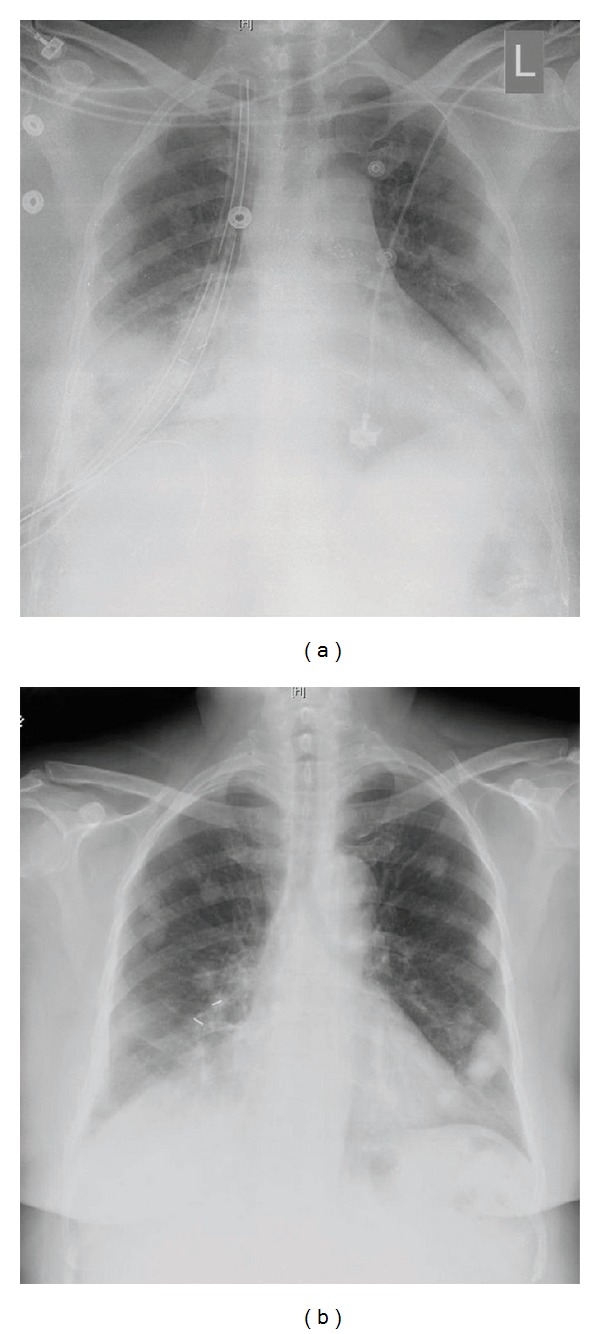
(a) CXR taken on postoperative day 1 which shows significant resolution of the fluid collection from the right hemithorax; (b) CXR taken on post-operative day 16 which shows complete resolution of the fluid collection from the right hemithorax.

During her current presentation, her physical examination was remarkable for oxygen saturation of 98% on 2 L of nasal cannula, absence of jugular venous distension and any lymphadenopathy, the presence of a 2 × 3 cm irregular and mobile mass in her right breast, clear breath sounds in the left lung but audible breath sounds only in the apex of the right lung, and dullness to percussion up to two-thirds of the right lung field. The abdomen was benign, and there was no evidence of pedal edema. Her labs were significant for leukocytosis (15.7 k/mm^3^), thrombocythemia (437 k/mm^3^), and mild hyponatremia (133 mEq/L). Her chest X-ray (CXR) revealed a complete white-out of the right side when compared to her previous CXR (Figures 1(a) and 1(b)). A bedside ultrasound showed a complex hypoechoic space with no clear pocket for thoracentesis. A chest CT with contrast showed the entire right hemithorax filled with fluid and an irregular mass (Figures 2(b) and 2(c)). Placement of a pigtail catheter resulted in drainage of 1500 cc of sanguinous fluid with RBC of 1.5 million/mm^3^, glucose of 28, LDH of 966 IU/mL, and a total protein of 5.6 g/dL. The fluid cytology showed blood and inflammatory cells, but no malignant cells were detected. Flow cytometry did not reveal cells with markers characteristic of lymphoma. During the next 24 hours, the chest tube drained 2000 cc of sanguinous fluid, and her hemoglobin dropped from 12.4 to 11 g/dL. The patient reported a significant improvement in her symptoms. However, a repeat CXR showed a significant amount of fluid in the lower lung field of the right lung. A repeat chest CT was suspicious of a bleeding vessel from a necrotic mass in the right middle lobe (Figures 3(a), 3(b), and 3(c)). She was taken to the OR and underwent a right-sided video-assisted thoracoscopic surgery (VATS) which was converted to right-sided open thoracotomy. During the procedure, approximately 2500 cc of sanguinous fluid was removed from the hemithorax, and the right middle lobe was removed after a large bleeding mass was noted in it. The intraoperative frozen section pathology revealed a spindle cell mass. After partial decortication, the remaining parts of the right lung was successfully reinflated. The pathological examination of the excised lung mass revealed two foci of metastasizing cellular leiomyoma with minimal cellular atypia and occasional mitoses. No necrosis or lymphovascular invasion was noted, and the bronchial and vascular margins were negative. The foci showed spindle cells and stained positive for smooth muscle actin (SMA), vimentin, desmin cytokeratin 7 (CK 7) (Figures 4(a)–4(f)). The pulmonary lesions were found to be histologically similar to uterine leiomyoma and did not meet the histological criteria for sarcoma. Follow-up CXRs taken on postoperative days 1 and 16 showed gradual but complete resolution of the right-sided mass (Figures 5(a) and 5(b)).

## 3. Discussion

BML has been observed to express estrogen and progesterone receptors and is known to regress during pregnancy, after menopause and with selective estrogen modulator therapy [17]. This may explain the relative paucity of medical literature on BML in postmenopausal women. Lungs are the most common site of metastasis for BML, but pulmonary BML in postmenopausal women has been described only in a handful of cases [18–22]. A majority of premenopausal women with pulmonary BML have multiple lung nodules, and a minority of patients have solitary lung nodules [23]. The monoclonal origin of BML has been established by cytogenetic studies [24]. It has been postulated that pulmonary BML may specifically arise from distinct subset of cells in the uterine leiomyoma with characteristic chromosomal aberrations of 19q and 22q deletions [25]. Most patients with pulmonary BML are asymptomatic and are diagnosed when pulmonary lesions are incidentally found on imaging. It is rarely associated with another malignancy, but association with primary lung cancer has been documented [26]. In the absence of tissue diagnosis, these lesions may be differentiated from primary lung cancer due to their low FDG activity on PET scans [27]. However, in the presence of a known tissue diagnosis of pulmonary benign leiomyoma, if some FDG avid lesions are noted amongst other non-FDG lesions on PET, leiomyosarcoma should be suspected [28, 29]. Detection of microRNA 221 (miR-221) has been recently shown to help differentiate leiomyoma from leiomyosarcoma [30].

Management of pulmonary BML depends on the extent of disease, presence of estrogen and progesterone receptors on the tumor, and the age of the patient. Preoperative treatment with GnRH agonists has been utilized to reduce tumor size and bleeding in women with uterine leiomyoma, but its role in treating pulmonary BML in postmenopausal women has not been clearly established. Most post-menopausal women with limited disease and with positive expression of estrogen receptors respond favorably to hormonal therapy with selective estrogen receptor modulator and aromatase inhibitors [31]. A combination of GnRH agonists and aromatase inhibitors has been shown to be effective in reducing tumor burden in patients with metastatic leiomyoma [32]. However, surgical resection may become necessary if the tumor results in complications such as the development of a hemothorax. Notably, patients with pulmonary lesions which are not resected and are not treated with hormonal therapy may develop cysts in the lung which may destroy the entire lung cavity [33]. Pulmonary embolism has been reported to be associated with pulmonary BML, and anticoagulation may be reasonable, especially in patients with heavy tumor burden [34]. 

Hemothorax is a rare manifestation of pulmonary BML. There has been one report of pulmonary BML noted in a nodule in the pulmonary artery which was successfully resected [35]. Pleural collection of blood due to pulmonary BML may also get superinfected and present as empyema [36]. This case illustrates the need to consider pulmonary BML as a possible etiology of hemothorax in post-menopausal women.
